# Assessment of the factors affecting the clinical outcomes of infection and safety of vaccines against SARS-CoV-2 among Egyptian patients

**DOI:** 10.1186/s12879-024-10362-8

**Published:** 2025-03-19

**Authors:** Amira A. Zidan, Ahmed Yousef Jad, Nermine H. Zakaria, Hazem M. El-Hariri, Maged El-Setouhy

**Affiliations:** 1 Department of Clinical Pharmacy, El-Beheira Health Affairs, Ministry of Health, Damanhour, Egypt; 2 Department of Chest Disease, Faculty of Medicine, Alexandria University, Alexandria, Egypt; 3 Department of Clinical Pathology, Faculty of Medicine, Alexandria University, Alexandria, Egypt; 4 Department of Community Medicine, National Research Centre, Cairo, Egypt; 5 Department of Family and Community Medicine, Faculty of Medicine, Jazan University, Jazan, Kingdom of Saudi Arabia; 6 Department of Community, Environmental and Occupational Medicine, Faculty of Medicine, Ain Shams University, Cairo, Egypt

**Keywords:** ChAdOx1 nCoV-19, AstraZeneca, Ad26.COV2.S, Johnson & Johnson, Moderna, BNT162b2 mRNA (Pfizer-BioNTech) CoronaVac (Sinovac), BBIBP-CorV / WIBP-CorV (sinopharm), Gam-COVID-Vac (Sputnik), COVID-19 vaccines, Viral vector, Messenger RNA (mRNA), Inactivated virus vaccines, Efficacy, Safety

## Abstract

**Background:**

Understanding the factors that influence clinical outcomes of COVID-19 and the safety of various vaccines is important to inform public health strategies, particularly in diverse communities. This study aimed to assess the factors affecting the clinical outcomes of COVID-19 and vaccination safety among the Egyptian population.

**Methods:**

In a retrospective study, we examined 1597 patients who tested positive for COVID-19. Among them, 1280 patients had received the vaccination, while 317 patients had not. We collected data from medical records, which included clinical characteristics, comorbidities, disease severity, type of vaccination, and adverse hematological effects postvaccination. We calculated the relative risk, odds ratio, and 95% confidence intervals (CIs).

**Results:**

Among the 1,597 COVID-19 cases, 74.1% were mild, 24.8% were moderate, and 1.1% were severe. Significant factors for moderate/severe cases included male sex (RR 0.78, 95% CI 0.64–0.95), cardiovascular diseases (RR 1.86, 95% CI 1.32–2.64), respiratory diseases (RR 1.40, 95% CI 1.08–1.82), diabetes mellitus (RR 1.41, 95% CI 1.07–1.86), and previous COVID-19 infection (RR 1.22, 95% CI 1.02–1.46). Vaccination reduced the severity risk, with BBIBP-CorV (Sinopharm) showing a significant protective effect (OR 0.78, 95% CI 0.62–0.98). Clinical presentations varied, with 97.6% having an oxygen saturation ≥ 92%. Logistic regression indicated that male sex and BBIBP-CorV (Sinopharm) vaccination were protective factors. Linear regression revealed that the male sex increased hemoglobin and leucocyte counts, whereas BBIBP-CorV (Sinopharm) decreased leucocyte and platelet counts.

**Conclusion:**

Vaccination, particularly with BBIBP-CorV (Sinopharm), significantly reduces COVID-19 severity among Egyptians, despite various clinical presentations and hematological effects.

**Clinical trial number:**

Not applicable.

## Introduction

The COVID-19 pandemic caused by the SARS-CoV-2 virus has significantly impacted daily life, the economy, and global health. As of 21 July 2024, the World Health Organization (WHO) recorded 7,054,891 deaths out of 775,731,698 cases. By December 31, 2023, 5.47 billion doses of COVID-19 vaccines had been administered worldwide [1].

Scientists from around the world have worked to develop vaccines that could combat COVID-19. The American College of Clinical Pharmacology supported the US FDA in approving these vaccines based on scientific evidence for COVID-19 prevention. As a result of widespread vaccination, mortality rates have decreased, and people have been protected against SARS-CoV-2, including the Omicron variant [2–4]. Scientists have used various strategies, including protein subunits, viral vectors[ChAdOx1 nCoV-19 (AstraZeneca)), Ad26.COV2.S ( Johnson & Johnson)), and Gam-COVID-Vac (Sputnik) )], messenger RNA (mRNA) [BNT162b2 mRNA (Pfizer-BioNTech), mRNA-1273mRNA (Moderna)], and inactivated virus techniques such as [CoronaVac (Sinovac)and BBIBP-CorV / WIBP-CorV (Sinopharm)]; these vaccines have shown positive outcomes and neutralizing antibodies that offer protection against the disease. However, COVID-19 immune responses tend to decline within two months [5, 6]. On the other hand, a systematic review revealed a nonsignificant difference in immunity between patients who recovered and those who were fully vaccinated against COVID-19 [7]. Recently, a systematic review showed that COVID-19 vaccines successfully reduce the incidence of infection, severity, hospitalization, and mortality, and studies assessing different ethnicities are recommended that do not have enough data about different variants; for example, in Egypt, further evaluation is necessary to determine the effectiveness of these vaccines against other recently emerged variations. Additionally, few studies are available on the efficacy/effectiveness of the second dose of the Gam-COVID-Vac (Sputnik), Novavax, CoronaVac, or ChAdOx1 nCoV-19(AstraZeneca) vaccine [8]. Additionally, a systematic review reported the need to evaluate the effectiveness of previous infections in preventing the recurrence of COVID-19 according to the status of vaccination [9]. The recommended interval between the first and second doses of mRNA-1273mRNA (Moderna) is 4–8 weeks [10], that of BNT162b2 mRNA (Pfizer-BioNTech) BioNTech be 3–4 weeks [11], and that of ChAdOx1 nCoV-19(AstraZeneca) be 8–12 weeks [12]. CoronaVac (Sinovac) is 2–4 weeks [13], that of BBIBP-CorV (Sinopharm) is 3–4 weeks [14], a single-dose regimen of Ad26.COV2.S( Johnson & Johnson) [15], that of Gam-COVID-Vac (Sputnik) is 21 days [16], and a booster dose may be considered 4–6 months after the primary vaccination series is completed.

They have shown efficacy in preventing severe illness and death associated with COVID-19, but they might also cause adverse reactions. The most common adverse symptoms from vaccination are nonlife-threatening (e.g., injection site pain, fatigue, dizziness, headache, fever, and bone and muscle pains) [17–20], and only 10% of the subjects suffer from severe side effects [20]. Severe side effects, such as immune thrombotic thrombocytopenia, can occur after vaccination with the ChAdOx1 viral vector for the treatment of COVID-19 [21, 22]. The two adenoviral vector vaccines used are ChAdOx1 nCoV-19 (Astra-Zeneca) and Ad26.COV.2. S ( Johnson & Johnson)) reported similar symptoms and mortality risks in patients with vaccine-induced thrombotic thrombocytopenia (VITT) [23], possibly because VITT ad-vector vaccines interact with platelets and/or PF4 [24]. A self-controlled case series revealed that the most common hematologic adverse event was lymphadenopathy, followed by anemia, which occurred in 44 patients (86.27%) after more than four weeks, and thrombocytopenia. In 31 patients (11.57%), thrombocytopenia and immune thrombocytopenia were the most common adverse events and the most common causes of hospitalization post-COVID-19 vaccination [25, 26]. A large-scale study on people with homogeneous same-country conditions was advised by a systematic review and meta-analysis that evaluated adverse events following COVID-19 vaccination with certain limitations as an analysis of the available data with heterogeneous individuals [26]. Most studies have evaluated the adverse effects of vaccines via questionnaires or clinical trials but are important for determining the rate of adverse events in communities with heterogeneous natures; however, few studies have considered side effects by comparing vaccinated participants with unvaccinated participants.

Therefore, we conducted this study to assess the factors affecting the clinical outcomes of COVID-19 and the safety of vaccination among the Egyptian population, we examined the impact of vaccination on hematology parameters (platelets, white blood cells, and hemoglobin) in the Egyptian population and to assess the safety of different vaccine strategies on platelet counts in vaccinated individuals. Additionally, we sought to evaluate disease severity among partially vaccinated, fully vaccinated, and booster doses.

## Methods

We conducted a cohort study from December 2021 to September 2022 to collect all recorded anonymous data from patients admitted to the outpatient clinic of Alexandria University Hospital who tested positive for COVID-19 through reverse transcriptase‒polymerase chain reaction (RT‒PCR) nasal and pharyngeal swab tests. We collected data about the severity of the disease, comorbidities, descriptive data of the patients, and adverse hematological effects. We classified 1597 COVID-19 patients into two groups based on their vaccination status: 1280 vaccinated patients and 317 unvaccinated patients. We then divided the patients into two groups based on the severity of the disease, as per the WHO criteria: the first group consisted of 1183 mild cases, which had no evidence of hypoxia or viral pneumonia. The second group included 414 moderate/severe cases. These patients presented signs of pneumonia, such as cough, fever, dyspnea, and fast breathing, but did not have severe pneumonia and had oxygen saturation (SpO2) levels of 90% or higher while breathing normal room air. Severe cases presented clinical signs of pneumonia, such as cough, fever, dyspnea, and fast breathing, and met one of the following criteria: respiratory rate greater than 30 breaths/min, severe respiratory distress, or SpO2 levels less than 90% while breathing normal room air [1, 27]. According to the CDC and WHO, vaccine doses are categorized as partial, full, or booster based on the number of doses taken by patients.

### Data analysis

The collected data were coded, tabulated, and statistically analyzed via IBM SPSS statistics (Statistical Package for Social Sciences) software version 28.0, IBM Corp., Chicago, USA, 2021. The normality of the quantitative data was tested via the Shapiro‒Wilk test; normally distributed data are presented as the means ± SDs (standard deviations), and the data were subsequently compared via an independent t-test. Qualitative data are presented as numbers and percentages and were compared via the chi-square test and Fisher’s exact test for variables with small expected numbers. Logistic and linear regressions were used to determine the independent factors affecting the study outcomes. A *p*-value ≤ 0.050 was considered to indicate statistical significance; otherwise, the difference was considered not significant.

## Results

Among the 1597 studied COVID-19-infected patients, 1183 (74.1%) had mild disease, 396 (24.8%) had moderate disease, and 18 (1.1%) had severe disease.

**Table 1:** Factors associated with COVID-19 severity

Variables		All cases	Severity		*p*-value	Relative risk (95% CI)
			Moderate/Severe	Mild		
Age (years)		35.5 ± 12.8	35.8 ± 13.3	35.3 ± 12.6	^0.569	Not applicable
Sex	Male	459	99 (21.6%)	360 (78.4%)	#0.012*	0.78 (0.64–0.95)
	Female	1138	315 (27.7%)	823 (72.3%)		Reference
Hypertension	Yes	181	67 (37.0%)	114 (63.0%)	#<0.001*	1.51 (1.22–1.87)
	No	1416	347 (24.5%)	1069 (75.5%)		Reference
Cardiovascular diseases	Yes	38	18 (47.4%)	20 (52.6%)	#0.002*	1.86 (1.32–2.64)
	No	1559	396 (25.4%)	1163 (74.6%)		Reference
Respiratory diseases	Yes	116	41 (35.3%)	75 (64.7%)	#0.016*	1.40 (1.08–1.82)
	No	1481	373 (25.2%)	1108 (74.8%)		Reference
Diabetes mellitus	Yes	101	36 (35.6%)	65 (64.4%)	#0.021*	1.41 (1.07–1.86)
	No	1496	378 (25.3%)	1118 (74.7%)		Reference
Autoimmune diseases	Yes	48	15 (31.3%)	33 (68.8%)	#0.393	1.21 (0.79–1.86)
	No	1549	399 (25.8%)	1150 (74.2%)		Reference
Hyperthyroidism	Yes	17	1 (5.9%)	16 (94.1%)	§ 0.090	0.23 (0.03–1.51)
	No	1580	413 (26.1%)	1167 (73.9%)		Reference
Malignancy	Yes	13	2 (15.4%)	11 (84.6%)	§ 0.534	0.59 (0.16–2.12)
	No	1584	412 (26.0%)	1172 (74.0%)		Reference
Neurological diseases	Yes	10	5 (50.0%)	5 (50.0%)	§ 0.138	1.94 (1.04–3.63)
	No	1587	409 (25.8%)	1178 (74.2%)		Reference
Chronic kidney disease	Yes	11	3 (27.3%)	8 (72.7%)	§ 0.999	1.05 (0.40–2.77)
	No	1586	411 (25.9%)	1175 (74.1%)		Reference
Chronic liver disease	Yes	12	3 (25.0%)	9 (75.0%)	§ 0.999	0.96 (0.36–2.58)
	No	1585	411 (25.9%)	1174 (74.1%)		Reference
Hypothyroidism	Yes	9	4 (44.4%)	5 (55.6%)	§ 0.249	1.72 (0.83–3.59)
	No	1588	410 (25.8%)	1178 (74.2%)		Reference
Pregnancy	Yes	12	5 (41.7%)	7 (58.3%)	§ 0.203	1.61 (0.82–3.17)
	No	1585	409 (25.8%)	1176 (74.2%)		Reference
Lactation	Yes	6	1 (16.7%)	5 (83.3%)	§ 0.999	0.64 (0.11–3.85)
	No	1591	413 (26.0%)	1178 (74.0%)		Reference
Previous COVID-19 infection	Yes	421	126 (29.9%)	295 (70.1%)	#0.029*	1.22 (1.02–1.46)
	No	1176	288 (24.5%)	888 (75.5%)		Reference

Data are presented as Mean ± SD or number (%). Percentages were taken for severity outcome from a total of exposures (rows).^Independent t-test. #Chi square test. §Fisher’s exact test. *Significant (≤ 0.050)

Table (1) showed that patients with moderate or severe COVID-19 infection were significantly less likely to be male and had cardiovascular disease, hypertension, respiratory disease, diabetes mellitus, and previous COVID-19 infection significantly more frequently.

**Table 2:** Impact of Vaccination and Different Vaccine Types on COVID-19 Infection Outcomes

Variables		All cases	Severity		*p*-value	Odds ratio (95% CI)
			Moderate/Severe	Mild		
Vaccine status	None	317	81 (25.6%)	236 (74.4%)	#0.804	Reference
	Partial	226	64 (28.3%)	162 (71.7%)		1.11 (0.84–1.47)
	Full	981	252 (25.7%)	729 (74.3%)		1.01 (0.81–1.25)
	Booster	73	17 (23.3%)	56 (76.7%)		0.91 (0.58–1.44)
One or more doses of any vaccine	Yes	1280	333 (26.0%)	947 (74.0%)	#0.866	1.02 (0.83–1.26)
	No	317	81 (25.6%)	236 (74.4%)		Reference
CoronaVac (Sinovac)	Yes	450	121 (26.9%)	329 (73.1%)	#0.581	1.05 (0.88–1.26)
	No	1147	293 (25.5%)	854 (74.5%)		Reference
BBIBP-CorV (Sinopharm)	Yes	308	65 (21.1%)	243 (78.9%)	#0.032*	0.78 (0.62–0.98)
	No	1289	349 (27.1%)	940 (72.9%)		Reference
ChAdOx1 nCoV-19 (AstraZeneca)	Yes	307	88 (28.7%)	219 (71.3%)	#0.223	1.13 (0.93–1.39)
	No	1290	326 (25.3%)	964 (74.7%)		Reference
BNT162b2 mRNA (Pfizer-BioNTech)	Yes	141	41 (29.1%)	100 (70.9%)	#0.371	1.14 (0.86–1.49)
	No	1456	373 (25.6%)	1083 (74.4%)		Reference
Ad26.COV2.S ( Johnson & Johnson)	Yes	38	7 (18.4%)	31 (81.6%)	#0.285	0.71 (0.36–1.38)
	No	1559	407 (26.1%)	1152 (73.9%)		Reference
mRNA-1273mRNA (Moderna)	Yes	24	7 (29.2%)	17 (70.8%)	#0.715	1.13 (0.60–2.11)
	No	1573	407 (25.9%)	1166 (74.1%)		Reference
Gam-COVID-Vac (Sputnik)	Yes	12	4 (33.3%)	8 (66.7%)	§ 0.521	1.29 (0.58–2.88)
	No	1585	410 (25.9%)	1175 (74.1%)		Reference
Different vaccines	Yes	947	237 (25.0%)	710 (75.0%)	#0.323	0.92 (0.78–1.09)
	No	650	177 (27.2%)	473 (72.8%)		Reference
*Duration since last dose (weeks) (among vaccinated)*						
All vaccinated (total = 1280-333-947)		23.3 ± 13.7	21.2 ± 12.1	24.1 ± 14.2	^0.001*	Not applicable
CoronaVac (Sinovac) (total = 450-121-329)		21.7 ± 13.3	20.0 ± 12.0	22.4 ± 13.7	^0.103	Not applicable
BBIBP-CorV (Sinopharm) (total = 308-65-243)		24.1 ± 14.8	21.0 ± 11.5	24.9 ± 15.5	^0.059	Not applicable
ChAdOx1 nCoV-19 (AstraZeneca) (total = 307-88-219)		24.7 ± 11.4	22.7 ± 9.5	25.5 ± 12.0	^0.058	Not applicable
BNT162b2 mRNA (Pfizer-BioNTech) (total = 141-41-100)		23.5 ± 15.6	22.2 ± 16.2	24.1 ± 15.4	^0.521	Not applicable
Ad26.COV2.S ( Johnson & Johnson) (total = 38-7-31)		24.1 ± 17.5	19.4 ± 17.8	25.2 ± 17.5	^0.435	Not applicable
mRNA-1273mRNA (Moderna) (total = 24-7-17)		21.4 ± 14.7	14.2 ± 11.6	24.3 ± 15.2	^0.130	Not applicable
Gam-COVID-Vac (Sputnik) (total = 12-4-8)		29.4 ± 10.7	33.4 ± 10.0	27.4 ± 11.1	^0.389	Not applicable

Data are presented as Mean ± SD or number (%). Percentages were taken for severity outcomes from the total exposures (rows). Vaccine type was considered based on the last dose if the case had received multiple types.^Independent t-test. #Chi square test. §Fisher’s exact test. *Significant (≤ 0.050)

Table (2) showed BBIBP-CorV (Sinopharm)’s notable decrease in the risk of moderate/severe COVID-19 infection (*p* = 0.032), with an odds ratio of 0.78 (95% CI: 0.62–0.98). Individuals with moderate/severe infections had a much shorter time since their last vaccine dose than those with mild infections (*p* = 0.001).

**Table 3:** Frequency of Signs and Symptoms Presented by COVID-19 Patients

Variables			All cases (Total = 1597)	
			*n*	%
Temperature (°C)		< 38.4	1489	93.2%
		≥ 38.4	108	6.8%
Oxygen saturation (%)		< 92	39	2.4%
		≥ 92	1558	97.6%
Headache			1232	77.1%
Cough			1163	72.8%
Sore throat			979	61.3%
Rhinorrhea			788	49.3%
Dyspnea			409	25.6%
Diarrhea			391	24.5%
Abdominal pain			390	24.4%
Vomiting			220	13.8%
Chest pain			34	2.1%
Nausea			16	1.0%
Shivering			10	0.6%
Severity	Mild		1183	74.1%
	Moderate		396	24.8%
	Severe		18	1.1%
			Total = 1597	
Anemia (Male: <12.5 gm/dL Female: <11.5 gm/dL)			272	17.2
Leucopenia (< 4.5 × 10^3^/mL)			298	18.8
leukocytosis (> 11.0 × 10^3^/mL)			65	4.1
Thrombocytopenia (< 150.0 × 10^3^/mL)			56	3.5
Thrombocytosis (> 450.0 × 10^3^/mL)			36	2.3
			Mean ± SD	Range
Hemoglobin (gm/dL)			12.8 ± 1.5	4.6–17.0
Leucocytes (x10^3^/mL)			6.5 ± 2.5	1.9–30.2
Platelets (x10^3^/mL)			261.5 ± 78.2	45.0–635.0

Table (3) The clinical presentation of the study patients. The oxygen saturation ≥ 92, headache, cough, and sore throat were the most frequent clinical presentations among the study cases.

**Table 4:** Logistic regression model for independent factors affecting moderate/severe disease severity

Factors	β	SE	*p*-value	OR (95% CI)
Male gender	-0.65	0.13	< 0.001*	0.52 (0.41–0.67)
Cardiovascular diseases	0.84	0.34	0.014*	2.32 (1.18–4.56)
BBIBP-CorV (Sinopharm) vaccine any dose	-0.32	0.16	0.041*	0.72 (0.53–0.99)
Vaccine status grades (no vaccine was a reference)			< 0.001*	
Vaccine status (partial)	-0.71	0.15	< 0.001*	0.49 (0.36–0.66)
Vaccine status (full)	-0.85	0.08	< 0.001*	0.43 (0.36–0.50)
Vaccine status (booster)	-0.94	0.29	< 0.001*	0.39 (0.22–0.68)

β: Regression coefficient. SE: Standard error. OR: odds ratio. CI: Confidence interval. *Significant

Table (4) showed that all the studied demographic, medical, and vaccine factors were studied via logistic regression. Cardiovascular disease (CVD) was a significant independent risk factor for moderate/severe-grade OR (95% 2.32 (1.18–4.56)), whereas male sex and receiving any dose of the BBIBP-CorV (Sinopharm) vaccine as well as vaccine status (partial, full, and booster) were significant independent protective factors against moderate/severe-grade disease. The protective effect against no vaccination was lowest for partial vaccination, followed by full vaccination, and highest for booster vaccination.

**Table 5:** Linear regression models for independent factors affecting hemoglobin, leukocytes, and platelets

Factors	β	SE	*p*-value	95% CI	R2
Hemoglobin (gm/dL) in all cases					
Constant	12.40	0.04	< 0.001*	12.31–12.49	20.4%
Male gender	1.52	0.08	< 0.001*	1.35–1.68	
Chronic kidney disease	-1.09	0.51	0.034*	-2.10–-0.08	
Leucocytes (x10^3^/mL) among all cases					
Constant	6.52	0.09	< 0.001*	6.34–6.71	1.1%
Male gender	0.33	0.16	0.043*	0.01–0.65	
BBIBP-CorV (Sinopharm), one or more doses	-0.57	0.17	0.001*	-0.90–-0.23	
Platelets (x10^3^/mL) among all cases					
Constant	272.46	2.46	< 0.001*	267.64–277.28	3.7%
Male gender	-25.45	4.27	< 0.001*	-33.82–-17.07	
Chronic liver disease	-80.81	22.26	< 0.001*	-124.48–-37.15	
BBIBP-CorV (Sinopharm), one or more doses	-15.99	4.89	0.001*	-25.59–-6.39	
Platelets (x10^3^/mL) among vaccinated					
Constant	247.56	4.26	< 0.001*	239.20–255.93	6.8%
Male gender	-27.48	4.53	< 0.001*	-36.37–-18.59	
Chronic liver disease	-54.33	24.27	0.025*	-101.94–-6.72	
BBIBP-CorV (Sinopharm), one or more doses	-14.47	4.76	0.002*	-23.81–-5.12	
Duration since the last dose of any vaccine (weeks)	0.99	0.15	< 0.001*	0.70–1.28	

β: Regression coefficient. SE: Standard error. CI: Confidence interval. *significant. R2: Coefficient of determination

Table (5) shows that all the studied demographic, medical, and vaccine factors were studied via linear regression. Among all vaccinated patients, male sex was a significant independent factor associated with increased hemoglobin levels, whereas chronic kidney disease was a significant independent factor associated with decreased hemoglobin levels. This model explained 20.4% of the hemoglobin variability among all vaccinated patients. Among all vaccinated cases, male sex was a significant independent factor that increased the leucocyte count, whereas receiving one or more doses of BBIBP-CorV (Sinopharm) was a significant independent factor that decreased the leucocyte count. This model explained 1.1% of the leucocyte variability among all vaccinated patients and among vaccinated patients. Among all vaccinated patients, male sex, chronic kidney disease, and receiving one or more doses of BBIBP-CorV (Sinopharm) were significant independent factors that decreased the platelet count. While duration since the last dose of any vaccine was a significant independent factor that increased the platelet count among vaccinated patients, these models explained 3.7% and 6.8% of the platelet variability among all patients and vaccinated patients, respectively. The independent effects of receiving the BBIBP-CorV (Sinopharm) vaccine on leucocytes and platelets, and the duration since the last dose of any vaccine on platelets represent the side effects and safety of vaccines.

## Discussion

Numerous studies have analyzed the risk factors for and characteristics of COVID-19 and the efficacy of vaccines worldwide. However, these factors vary across geographical regions and cannot be generalized globally because of differences in responses to viruses and vaccinations. Therefore, we conducted a study to assess the clinical characteristics and risk factors affecting the severity of the disease and the safety of vaccination in Egyptian patients. Our data revealed that sex; comorbid diseases such as sex; cardiovascular disease (CVD); diabetes mellitus; hypertension; respiratory disease; and previous COVID-19 infection were risk factors for severe disease.

Our study found that gender is a risk factor for the severity of COVID-19. Specifically, severe cases were less frequent in males than in females, and there were significant independent protective factors against having a moderate/severe grade according to logistic regression. These results may agree with the findings of several studies in a systematic review and meta-analysis in 2022, which revealed that long COVID-19 duration is associated with female sex [28]. , and a prospective study reported an increased risk of hospitalization in women compared with men [29]. Additionally, in the compartment model conducted by Doerre A. and Doblhammer G., sex differences were greater among women than among men of working age. In contrast, in old individuals, the death rate of males is twice that of women in all age categories [30]. These findings support our finding that working age increases the risk in females, whose mean age was 35.5 ± 12.8 years in our study. On the other hand, previous studies reported that males were at risk of severe disease [31, 32]. , Other cohort studies reported that the mortality rate was lower in females than in males [33].

We found that cardiovascular disease (CVD) in patients with moderate to severe COVID-19 was a significant independent risk factor for severe disease outcomes. This is consistent with findings from a previous systematic review, which indicated that CVD is associated with an increased risk of mortality in COVID-19 patients [34].

We found that hypertension and diabetes mellitus were more common in patients with moderate/severe disease. A previous systematic review and meta-analysis revealed that hypertension was an independent risk factor for critical COVID-19 and mortality (1.82-fold and 2.17-fold, respectively) [32]. Another cohort study revealed that hypertension, diabetes mellitus, and coronary artery disease were risk factors for disease progression [31]. A recent century clinical study of 1.5 million patients with COVID-19 reported that hypertension and diabetes mellitus are associated with the severity of COVID-19 [35].

The respiratory disease risk factors in our study included both upper and lower respiratory conditions, such as asthma chronic sinusitis, and allergic rhinitis. Asthma was significantly more common in patients with moderate/severe COVID-19. A prospective study conducted by Finkas Lindsay K et al. among 41,282 patients with asthma revealed that asthma was an independent risk factor for COVID-19 hospitalization and was strongly associated with severe asthma [29]. On the other hand, a retrospective study of 1526 COVID-19 patients (asthmatic and non-asthmatic patients) revealed that an increased risk of hospitalization was not associated with asthma. There was no association between hospitalization linked to COVID-19 and the use of inhaled corticosteroids, whether administered systemically or not [36].

Our research observed that moderate to severe cases of COVID-19 are more common in individuals with a previous infection, This may be because they have comorbidities and risk factors for contracting COVID-19. In addition to other studies, a previous cohort study involving 341 patients reported that the risk of hospitalization and mortality was significantly lower in individuals who were previously infected or had been fully vaccinated against COVID-19 [37]. A cross-sectional study of 432 people found that 41% of them had antibodies as a result of previous COVID-19 infection [38]. Another retrospective study suggested that unvaccinated people who were previously infected with COVID-19 had some level of protection against reinfection, which decreased from 97% after two months to 72% after eighteen months [39]. Another study reported that protection against infection decreased from 68% at ≤ 6 months to 13% at > 6 months [40].

In our study, we evaluated the relationship between vaccination status (none, partial, full, and booster) and the severity of COVID-19 infections using univariate analysis and logistic regression Additionally, we assessed the impact of different vaccine types, including (CoronaVac (Sinovac), BBIBP-CorV (Sinopharm), ChAdOx1 nCoV-19(AstraZeneca), BNT162b2 mRNA (Pfizer-BioNTech), Ad26.COV2.S ( Johnson & Johnson), mRNA-1273mRNA (Moderna), and Gam-COVID-Vac (Sputnik) ), on infection severity. We found that the BBIBP-CorV (Sinopharm) vaccine significantly reduced the risk of severe COVID-19 infection. These findings suggest that individuals vaccinated with BBIBP-CorV (Sinopharm) were less likely to experience severe COVID-19 than those who did not receive this vaccine. On the other hand, other vaccines, such as CoronaVac (Sinovac), ChAdOx1 nCoV-19(AstraZeneca), BNT162b2 mRNA (Pfizer-BioNTech), Ad26.COV2.S ( Johnson & Johnson), mRNA-1273mRNA (Moderna), and Gam-COVID-Vac (Sputnik) did not significantly affect the severity of infections. This may be due to variability in population response or the need for larger sample sizes to detect differences. In logistic regression analysis, there was an independent significant protective effect of receiving any dose of the BBIBP-CorV (Sinopharm) vaccine against moderate/severe disease. Continuous monitoring and real-world effectiveness studies are essential to validate these observations.

The univariate analysis which may be affected by unknown confounders, showed a significant difference in the duration since the last vaccine dose between vaccinated individuals with moderate-to-severe infections and those with mild infections (*p* = 0.001). However, the multivariate analysis (Logistic regression), showed that the protective effect of vaccination was lowest for partial vaccination, followed by full vaccination, and was highest after receiving the booster dose. These findings indicate that the severity of COVID-19 decreases with more recent vaccinations. Previous research shows that the antibody titer significantly declines within one month following the second dose of the vaccine [41]. Previous studies have shown that vaccine effectiveness decreases with increasing duration after the second dose, and there is also a decrease in the effectiveness of the vaccine against hospitalization and mortality [39]. Other studies have reported that vaccine effectiveness differs according to the type of coronavirus (omicron vs. delta), rate of hospitalization, and need for oxygen therapy [40].

We evaluated the effect of vaccination status (vaccinated and unvaccinated) on a complete blood count (CBC) to identify potential variations in these parameters. Our linear regression analysis revealed that the male sex was associated with higher hemoglobin and leucocyte counts but lower platelet counts. Chronic kidney disease and chronic liver disease are linked to lower hemoglobin and platelet counts, respectively. The BBIBP-CorV (Sinopharm) vaccine is associated with lower leucocyte and platelet counts. Among vaccinated individuals, a longer duration since the last dose correlates with higher platelet counts. These findings indicate significant effects of sex, health conditions, and vaccination status on blood parameters in COVID-19 patients, in which we detected a decrease in leukocytes with partial or full doses of the BBIBP-CorV (Sinopharm) vaccine (570 white blood cells per microliter) and a decrease in the platelet count with a partial or full dose of the BBIBP-CorV (Sinopharm) vaccine (14,470 platelets per microliter). However, the duration since the last dose of any vaccine was a significant independent factor that increased the platelet count by 990 platelets per microliter per week among vaccinated patients. These insights highlight the need to consider these factors when assessing the hematologic health of COVID-19 patients.

Numerous studies have been conducted on the safety of COVID-19 vaccines, including BBIBP-CorV (Sinopharm)/CoronaVac (Sinovac), BNT162b2 mRNA(Pfizer-BioNTech), and ChAdOx1 nCoV-19(AstraZeneca). The results revealed that all the vaccines are safe. The most common side effects include pain at the injection site, flu-like symptoms, fatigue, and bone and muscle pain. However, these side effects were **less** common in recipients of BBIBP-CorV (Sinopharm)/CoronaVac (Sinovac) than in those receiving other vaccines [17]. One study showed that ChAdOx1 nCoV-19(AstraZeneca) had greater side effects than did BNT162b2 mRNA (Pfizer-BioNTech) and BBIBP-CorV (Sinopharm) [19]. Factors such as receiving the first dose, being female, and the type of vaccine (ChAdOx1 nCoV-19(AstraZeneca) versus BBIBP-CorV (Sinopharm)/CoronaVac (Sinovac)) were identified as significant predictors of adverse events, although serious reactions were rare. Overall, all vaccine types are considered safe for adults in Egypt [17, 18]. However, a cross-sectional study of 1246 COVID-19-vaccinated healthcare workers in Egypt reported that ChAdOx1 nCoV-19(AstraZeneca) has thrombotic complications and more common adverse effects than other vaccine types do [18].

A previous study (a self-controlled case series) showed that hematologic adverse events were more common in BNT162b2 mRNA (Pfizer-BioNTech)during the first vaccine dose, in which thrombocytopenia, or immune thrombocytopenia, was a cause of hospitalization for 38.71% of the patients in the range of 1–42 days who received BNT162b2 mRNA (Pfizer-BioNTech), followed by Oxford-ChAdOx1 nCoV-19(AstraZeneca) at 32.26% and mRNA-1273mRNA (Moderna) at 16.13% [25].

A systematic review found that cases of vaccine-related thrombosis caused by the BNT162b2 mRNA (Pfizer-BioNTech) and mRNA-1273mRNA (Moderna) vaccines occurred within 3 days of the last dose in three males. For the ChAdOx1 nCoV-19(AstraZeneca) vaccine, 58 cases occurred 7–16 days after vaccination, mostly in females, and the symptoms were thrombocytopenia with purpura or headache [42]. Additionally, 237 patients were found to have a higher frequency of thrombocytopenia in a systematic review and meta-analysis of adverse events following vaccination. This frequency was significantly greater with the viral vector vaccine (Oxford/ChAdOx1 nCoV-19(AstraZeneca) and Ad26.COV2.S( Johnson & Johnson)) than with the mRNA vaccines (BNT162b2 mRNA (Pfizer-BioNTech) and mRNA-1273mRNA (Moderna)) and inactivated virus vaccines. However, comparisons with the inactivated vaccine have been limited because of the significant difference in numbers between the two groups [26].

### Limitation

The study participants were recruited solely from Alexandria University Hospital in Egypt, which limits the generalizability of the findings.

## Conclusions

Our study showed that male patients and those who received BBIBP-CorV (Sinopharm) vaccination were less likely to develop moderate or severe COVID-19. On the other hand, cardiovascular disease (CVD) was a significant risk factor for moderate- to severe-grade illness. According to linear regression analysis, male gender was found to be a significant factor for increased hemoglobin levels and leucocyte counts among all patients and vaccinated patients. Moreover, receiving one or more doses of BBIBP-CorV (Sinopharm) significantly reduced the leucocyte count. Although, male gender, chronic kidney disease, and receiving one or more doses of BBIBP-CorV (Sinopharm) significantly decreased the platelet count, the duration since the last vaccine dose significantly increased the platelet count.

## Data Availability

The data supporting this study’s findings are available upon request from the corresponding author. The data are not publicly available due to privacy or ethical restrictions.
